# Assessment of Quality of Life (QoL) of Colorectal Cancer Patients using QLQ-30 and QLQ-CR 29 at King Abdulaziz Medical City, Jeddah, Saudi Arabia

**DOI:** 10.1155/2022/4745631

**Published:** 2022-05-17

**Authors:** Jumanah T. Qedair, Abdullah A. Al Qurashi, Saeed Alamoudi, Syed Sameer Aga, Alqassem Y. Hakami

**Affiliations:** ^1^Department of Basic Medical Sciences, College of Medicine, King Saud Bin Abdul Aziz University for Health Sciences (KSAU-HS), King Abdulaziz Medical City, Jeddah, Saudi Arabia; ^2^King Abdullah International Medical Research Centre (KAIMRC), National Guard Health Affairs (NGHA), Jeddah, Saudi Arabia

## Abstract

**Objective:**

We aimed to assess the quality of life (QoL) and its predictors in colorectal cancer (CRC) patients at King Abdulaziz Medical City, Jeddah.

**Methods:**

A total of 118 CRC patients at King Abdulaziz Medical City, a tertiary hospital in Jeddah, participated in this study. The participants were provided with the online questionnaire via WhatsApp by trained researchers and data collectors in February 2021. All participants were required to answer the three-section questionnaire comprising of (a) demographic data and a validated Arabic version of the European Organization for Research and Treatment of Cancer (EORTC) quality of life questionnaires, (b) a general version (QLQ-30), and (c) a CRC-specific version (QLQ-CR29).

**Results:**

Statistical analysis revealed that the most common comorbidity among the participants was diabetes mellitus (42.4%). In addition, the mean global health status was 63.91 ± 24.75. For the global health tool QLQ-C30, results exhibited that physical functioning [62.94 (30.04)] and social functioning [63.56 (31.95)] scored below the threshold, while the cognitive functioning scale scored the highest [74.86 (25.11)]. In addition, on the QLQ-C30 scales, fatigue and insomnia were distressing, with fatigue scoring the highest. For the disease-specific tool QLQ-CR29, it was found that for the symptom scale, urinary frequency and embarrassment scored the highest. Conclusion. The participants reported high global quality of life on both the EORTC QLQ-30 and QLQ-CR29 scales. This study identifies the factors and predictors that affect the quality of life of CRC patients in Saudi Arabia. Recognizing these factors and predictors may empower those patients to maintain positive perception towards the impact of colorectal cancer and improve their survival.

## 1. Introduction

Colorectal cancer (CRC) is the 3rd most common cancer and the 4^th^ leading cause of cancer-related deaths accounting for 1.4 million new cases and 700,000 deaths worldwide [1, 2]. Saudi Arabia is a low-risk country for CRC, yet recent reports show an increase in the incidence rate [3, 4].

CRC is the most common cancer among Saudi males and the third most common among Saudi females [3, 5, 6]. The Saudi cancer registry (SCR) reported an age-standardized incidence rate of 7.3 per 100,000 in 2011 [3, 7].

Cancer and its treatment carry a profound and long-lasting effect on the quality of life (QoL) of cancer survivors even years after the end of the treatment, not to mention the emotional impact on patients and their families [1]. QoL is a multidimensional concept that assesses multiple domains of patients with cancer including physical, role, emotional, cognitive, and social functioning and is used as an outcome measure for cancer patients [1, 3, 6]. Moreover, assessment of QoL in cancer patients provides insights on how the disease influences patients' lives and helps to fully evaluate the impact of the cancer experience and its treatment [1, 3, 8]. Accumulating evidence suggests an impaired QoL in CRC patients compared to the general population in the aspects of physical, emotional, and social functioning [1, 9, 10]. Multiple tools have been developed to assess the QoL in cancer patients, and importantly one such tool is the European Organization for Research and Treatment of Cancer (EORTC) quality of life questionnaires [3].

EORTC (QLQ C-30) is a structured multifaceted tool to assess QoL of patients with cancer and has been demonstrated to have adequate validity and reliability to evaluate outcomes of cancer patients across different countries [3, 11]. Given the high prevalence of the disease, concerns have been raised about how CRC affects QoL among patients in Saudi Arabia. There is a drought in literature regarding QoL of CRC patients in Saudi Arabia. As such, very little is known about how patients in Saudi Arabia endure a chronic and potentially life-threatening disease.

Therefore, this study aims to assess the QoL of CRC patients at the tertiary care hospital at King Abdulaziz Medical City, Jeddah, using the EORTC (QLQ C-30) assessment tool to provide a glimpse of the effect this burden has on life.

## 2. Methods

### 2.1. Subjects

Hundred and eighteen colorectal cancer (CRC) patients participated in this study with prior informed consent. The participation was through the invitation with full disclosure, and each participant was required to fill in the research questionnaire of this cross-sectional study. The study was conducted in King Abdulaziz Medical City (KAMC), a tertiary hospital in Jeddah, and it was specifically chosen because it provides a state-of-the-art practice of medical care services for the Saudi Arabian population in the Western Region. Also, the hospital has a designated center for cancer patients. The ethical approval was obtained from the Institutional Review Board (IRB) committee of the King Abdullah International Medical Research Center (KAIMRC).

### 2.2. Design Questionnaire

All eligible participants were contacted formally by the PI of the study through telephone and then provided with the online questionnaire via WhatsApp by trained researchers and data collectors. The participants answered the questions of a validated Arabic version questionnaire of the EORTC quality of life (QOL) questionnaires: a general version—QLQ-30 and a colorectal cancer specific version—QLQ-CR 29 (https://qol.eortc.org/questionnaires/) [11–13].

The online questionnaire comprised of three sections. The first section was about the demographic data that included participants' age, nationality, city, gender, marital status, level of education, employment status, monthly income, presence of comorbidities, and tumor location.

The second section of the questionnaire was the Arabic translated form of QLQ-C30 (version 3) which included 30 questions that assessed patients' overall health, functions, symptoms, and financial implications of the disease considering that each question ranges from “not at all” to “very much.”

The last section of the questionnaire was the QLQ-CR29 version that included disease symptom scales and functional scales and consisted of 29 questions that assessed body image, sexuality, and patients' future perspective. It aims to specifically evaluate the health-related quality of life among colorectal cancer patients. This questionnaire was provided in addition to the EORTC QLQ-C30 to investigate the treatment and its effects on patients' daily functioning.

The researchers contacted the EORTC quality of life group to obtain the Arabic version of the questionnaire and the scoring manual. Each response scale was recorded and transformed through a description to give a score between 0 and 100. Higher scores in functional scales indicate better functioning, whereas higher scores in symptom scales indicate worse functioning. For functional scales, subjects scoring <33.3% have problems; those scoring ≥66.7% have good functioning. For symptom scales/symptoms, subjects scoring <33.3% have good functioning; those scoring ≥66.7% have problems.

### 2.3. Inclusion and Exclusion Criteria

Following the IRB approval, patients' data was extracted from the BESTCare system in KAMC. The eligibility criteria included patients with a currently confirmed diagnosis of CRC from both genders and all ages. Patients who refused to participate and did not complete the questionnaire were excluded. Informed consent was provided with the questionnaire and obtained from all participants. Also, researchers checked patients' medical records to ensure the validity of the diagnosis, treatment method, stage of the disease, and patients' current status.

### 2.4. Statistical Analysis

Sociodemographic characteristics were presented as frequencies and percentages. The QLQ-C30 and C29 questionnaires were presented as the mean ± SD, 95% CI, percentage scoring <33.3, and percentage scoring ≥66.7. Scores were calculated as per EORTC QLQ-C30 scoring manual. Linear regression analysis was done to find out the factors predictive of global, functional, and symptoms scales. The analysis was performed in 95% confidence interval using the Statistical Package for Social Science (SPSS), version 24.0 (IBM, Armonk, NY, USA), and *p* value of ≤0.05 was considered statistically significant.

## 3. Results

Among the 118 participants, the age group of above 60 years old represented 47.5% of the total study sample. Among all cases, 64 (54.2%) were males, 58 (49.2%) were from Jeddah, 783 (70.3%) were married, only 24 (20.3%) were illiterate, 51 (43.2%) were retired, and 77 (65.3%) had colon tumor.

The most common comorbidity among the participants was diabetes mellitus (42.4%). The detailed demographic characteristic of the participants is presented in Table 1.

The mean global health status was 63.91 ± 24.75 (Table 2). For the global health tool QLQ-C30, only two of five functional scales scored below the threshold of ≥66.7% which were as follows: physical functioning 62.94 (30.04) and social functioning 63.56 (31.95); while as the scores for other three were as role functioning 67.51 (35.73), emotional functioning 69.00 (27.37), and cognitive functioning 74.86 (25.11).

For the symptom scale items, three out of nine symptoms had a good functioning which were as follows: nausea and vomiting 24.44 (27.79), dyspnea 22.60 (29.20), and financial difficulties 30.79 (34.63); while two were distressing: fatigue 46.14 (30.87) and insomnia 41.24 (36.38); and the rest of the four symptoms were mildly problematic as follows: pain 38.70 (31.42), appetite loss 35.59 (34.24), constipation 35.59 (34.52), and diarrhea 34.46 (34.30).

For the disease-specific measuring tool QLQ-CR29, the only item which had problems was identified as body image with a score of 33.71 (31.56); while other three had good scores as follows: anxiety 51.41 (36.37), weight 43.50 (34.73), and sexual interest (men) 38.54 (32.10).

For the symptom scale, two were identified as problematic ones, i.e., urinary frequency 41.67 (31.63) and embarrassment 40.93 (36.57); while five were found to have great functioning with scores less than 19 which are as follows: impotence 14.20 (22.06), urinary incontinence 14.69 (26.35), blood and mucus in stool 16.24 (23.41), fecal incontinence 17.72 (29.15), and dysuria 18.36 (27.42) (Table 2).

There was no significant difference between any of the demographic characteristics of patients and the global health scales. However, age and income were found to be significantly associated with the social functioning and cognitive functioning, respectively (*p* < 0.05) (Table 3). The analysis showed that old aged subjects had lesser functioning on social scales while higher income subjects had better functioning on the cognitive scales.

Our study results also revealed significant differences (*p* < 0.05) in the reported symptoms, i.e., nausea and vomiting and diarrhea across educational levels (Table 3), and income and financial difficulties across marital status, as well as tumor location. Married subjects with lower incomes complained of financial difficulties (*p* < 0.05).

The predictors related to participants' QoL as per QLQ- (CR29) are presented in Table 4. Significant associations were found between symptoms and various demographic characteristics of subjects: weight with marital status, urinary frequency with education level and employment, blood and mucus in stool with education level and income, dysuria with tumor location, sore skin with income, impotence with age, and dyspareunia with marital Status.

Additionally, married participants complained of intense symptoms of anxiety and dyspareunia (*p* < 0.05), while as those with higher education complained more of urinary frequency and blood and mucus in stool, and old aged subjects of impotence.

## 4. Discussion

Colorectal cancer (CRC) characterized by malignancy of colon or rectal lumen cells is one of the major solid cancers affecting humans [14]. Even though CRC incidence rates vary widely geographically, there has been an increasing trend on a yearly basis since last decade [15]. In 2018, it has become third most common and second most deadly cancer in the world, after lung and breast [16] in both genders. Furthermore, Western countries happen to have the highest incidences of CRC in comparison to Asian and Middle Eastern countries [7, 17–19].

In the Kingdom of Saudi Arabia (KSA), CRC ranks first among males (10.6%) and third in females (8.9%) [20]. In 2014, there were 1,347 cases of CRC which accounted for 11.5% of all newly diagnosed cases, posing a significant health risk to Saudi nationals [20, 21]. It has been reported that the median age for the development of CRC in the Saudi population is 60 years (95% CI: 57–61 years) for men and 55 years (95% CI: 53–58 years) for women [22]. Additionally, in Saudi Arabia, CRC tends to affect younger people more, and the 5-year survival rates have been reported to be lower (about 44.6%) than those expected for matching stages in other populations [7, 22].

In the current study, we attempted to evaluate the quality of life (QoL) among the CRC patients using the EORTC QLQ-C30 and QLQ-CR29 questionnaires. Additionally, we attempted to evaluate the functionality of the participants in dealing with the burden of progressive, chronic, and potentially fatal disease. This study aimed to identify the factors which affect the overall QoL and hence be of significance to healthcare professionals in further improving the CRC patients' survival.

In this study, we found that the CRC patients presented with a high level of functioning and quality of life, as evident from the high scores of the EORCT QLQ-C30 and QLQ-CR29 scales (Table 2). These results were in concordance with the study published by Almutairi et al. [6] and Alshehri et al. [23] which included patients from the central region of the country and reported higher functional scores on the QLQ-C30 scales. Additionally, in comparison to other similar studies in different geographical locations, the overall global health status and all the functional scores of our study were higher [24–26]. Since the study setting was in KAMC, it does reflect on the fact that cancer patients who have access to free and state-of-the- art healthcare services do tend to have a better QoL, as they feel less burdened by the disease financially as well as emotionally [24, 25].

Furthermore, 52.5% of our study participants were of age less than 60 years old (Table 1), which was similar to the previous studies but different from the report from Japan, where the majority of participants were above 70 years of age [24, 25, 27]. The higher percentage of young patients confirms the established fact that in Saudi Arabia CRC has a high prevalence among the younger people [7]. There are numerous risk factors which are associated with CRC among young individuals like smoking, high fat diet, low-fiber diet, sedentary lifestyle, less exercise, and higher consumption of fast food [6, 14].

Globally, for any type of cancer, age is regarded as the chief factor which affects the QoL of a patient. However, in our study we found that age was significantly associated with the limitation of social functioning only. These results were different than the ones reported by other studies [6, 28], which reported that the oldest age group (≥60 years) exhibited a tendency to score lowest in functional domains especially in physical functioning scales. This can be considered as the strength of our study in identifying that the healthcare facility provided at KAMC is at its best and provides a necessary alleviation of the quality of life factors. However, in our study we did find that age was related to impotence (*p* < 0.05).

In our study, we also found that cancer patients are concerned more about their body image, which scores the lowest in QLQ-CR29 scale (33.71). However, it was not found to be associated with any of the demographic characteristics of subjects. This is understandable, as most of our participants belonged to the younger age group (<60). It therefore presents a challenge for healthcare providers to mitigate the awareness about cancers in general and about CRC in particular, so that the disease is caught well in the beginning for the treatment to be effective and for the disease to be less crippling. Aga et al. have already reported that the awareness among health and allied students regarding colorectal cancer was low [29].

Therefore, there is a dire need of proactive, aggressive, and preventive medicine campaigns and educational programs to prepare the population for challenges posed by the increasing burden of cancer in the kingdom [20, 22, 29]. Additionally, urinary frequency scores highest among the QLQ-CR29 symptoms scales which was found to be associated with both education level and employment (*p* < 0.05) (Table 4). This highlights the dominant effect of education in identifying the most irritating symptom which affects the daily functioning of cancer patients.

We also found that marital status, education level, and income were the primary predictors of the quality of life among the CRC patients, as each of them was significantly associated with at least two symptoms of CRC (Tables 3 and 4). Married subjects with lower income were in particularly worried about the financial difficulties because of the burden of carrying cancer. These results were similar to the results reported by Almutairi et al. [6].

This study does have its own limitations. First, regarding the measuring tools, which lack the ability to measure the QoL before and after the treatment or intervention. Second, the study sample size is small for the generalization of the results for the whole population.

## 5. Conclusion

In conclusion, this study provides a glimpse into the QoL of CRC patients in our medical center and does sketch a good functioning of life among our participants as they reported a high global quality of life on both the EORTC QLQ-30 and QLQ-CR29 scales. Among the most distressing symptoms, fatigue and insomnia topped the list, and among predictors urinary frequency and blood and mucus in stool were found to be the most common symptoms. Predictors for the cognitive and social functioning were found to be age and income. This study reiterates the fact that the burden of carrying the cancer puts the patients at risk of poorer quality of life which needs to be mitigated well within social and private constraints to ease the suffering.

## Figures and Tables

**Table 1:** Demographic characteristics of the study population.

Characteristic	No	%
Age Below 40-year-old From 41- to 50-year-old From 51- to 60-year-old Above 60-year-old	10 20 32 56	8.5 16.9 27.1 47.5

Gender Male Female	64 54	54.2 45.8

City Jeddah Riyadh Others	58 1 59	49.2 0.8 50.0

Marital status Single Married Divorced Widow	5 83 9 21	4.2 70.3 7.6 17.8

Education level Noneducated Primary Secondary School High school University	24 18 13 26 37	20.3 15.3 11.0 22.0 31.4

Employment status No work Government Private Retired	42 20 5 51	35.6 16.9 4.2 43.2

Income/month Below 5000 SR Between 5 to 10 thousand SR Between 10 to 20 thousand SR More than 20 thousand SR	40 41 27 10	33.9 34.7 22.9 8.5

Presence of comorbid disease Diabetes mellitus Asthma Heart disease Hypertension	50 4 11 25	42.4 3.4 9.3 21.2

Tumor location Colon Rectum	77 41	65.3 34.7

**Table 2:** Mean score of all items in QLQ-C30 and QLQ-C29 (*n* = 118).

	Variables	N	No. of items	Mean (SD)	95% CI	N (%) scoring < 33.3	N (%) scoring ≥ 66.7
QLQ-C30	Global health status/QoL Global health status/QoL	118	2	63.91 (24.75)	59.40–68.42	7 (5.93)	64 (54.24)
	Functional scales Physical functioning Role functioning Emotional functioning Cognitive functioning Social functioning	118 118 118 118 118	5 2 4 2 2	62.94 (30.04) 67.51 (35.73) 69.00 (27.37) 74.86 (25.11) 63.56 (31.95)	57.46–68.41 61.00–74.03 64.01–73.99 70.28–79.44 57.73–69.38	15 (12.71) 17 (14.41) 12 (10.17) 7 (5.93) 14 (11.86)	64 (54.24) 77 (65.25) 59 (50.00) 91 (77.12) 72 (61.02)
	Symptom scales/items Fatigue Nausea and vomiting Pain Dyspnea Insomnia Appetite loss Constipation Diarrhea Financial difficulties	118 118 118 118 118 118 118 118 118	3 2 2 1 1 1 1 1 1	46.14 (30.87) 24.44 (27.79) 38.70 (31.42) 22.60 (29.20) 41.24 (36.38) 35.59 (34.24) 35.59 (34.52) 34.46 (34.30) 30.79 (34.63)	40.51–51.77 19.37–29.50 32.97–44.43 17.28–27.92 34.61–47.88 29.35–41.84 29.30–41.89 28.21–40.72 24.48–37.11	34 (28.81) 68 (57.63) 46 (38.98) 64 (54.24) 37 (31.36) 42 (35.59) 41 (34.75) 45 (38.14) 53 (44.92)	39 (33.05) 14 (11.86) 34 (28.81) 20 (16.95) 43 (36.44) 34 (28.81) 31 (26.27) 34 (28.81) 29 (24.58)

QLQ-CR29	Functional scales Body image Anxiety Weight Sexual interest (men) Symptom scales/items Urinary frequency Blood and mucus in stool Stool frequency Urinary incontinence Dysuria Abdominal pain Buttock pain Dry mouth Hair loss Taste Flatulence Fecal incontinence Sore skin Embarrassment Stoma care problems Impotence Dyspareunia	118 118 118 118 118 118 118 118 118 118 118 118 118 118 118 118 118 118 118 118 118	3 1 1 1 2 2 2 1 1 1 1 1 1 1 1 1 1 1 1 1 1	33.71 (31.56) 51.41 (36.37) 43.50 (34.73) 38.54 (32.10) 41.67 (31.63) 16.24 (23.41) 27.92 (27.23) 14.69 (26.35) 18.36 (27.42) 39.83 (34.66) 25.42 (34.23) 35.03 (30.77) 32.77 (35.67) 22.88 (30.41) 30.80 (34.50) 17.72 (29.15) 25.64 (33.52) 40.93 (36.57) 27.13 (29.33) 14.20 (22.06) 21.38 (35.26)	23.70–77.89 24.45–99.36 14.60–99.69 20.67–49.24 13.15–58.28 7.95–27.00 2.19–30.76 4.39–61.53 4.39–61.53 14.60–99.69 5.14–71.05 15.53–51.13 13.53–72.18 2.19–30.76 4.39–61.53 15.91–54.00 15.91–54.00 2.57–35.53 2.19–30.76 13.78–32.83 16.56–73.70	61 (51.69) 24 (20.34) 30 (25.42) 19 (16.10) 32 (27.12) 85 (72.03) 34 (28.81) 83 (70.34) 73 (61.86) 38 (32.20) 67 (56.78) 37 (31.36) 54 (45.76) 65 (55.08) 35 (29.66) 52 (44.07) 41 (24.75) 24 (20.34) 19 (16.10) 36 (30.51) 36 (30.51)	25 (21.19) 57 (48.31) 45 (38.31) 23 (19.49) 35 (29.66) 8 (6.78) 8 (6.78) 12 (10.17) 15 (12.71) 45 (38.14) 27 (22.88) 33 (27.97) 38 (32.20) 20 (16.95) 19 (16.10) 10 (8.47) 14 (11.86) 26 (22.03) 9 (7.63) 5 (4.24) 11 (9.32)

For functional scales, subjects scoring <33.3% have problems; those scoring ≥66.7% have good functioning. For symptom scales/symptoms, subjects scoring <33.3% have good functioning; those scoring ≥66.7% have problems. For functional scales, higher scores indicate better functioning. For symptom scales, higher scores indicate worse functioning.

**Table 3:** Predictors of quality of life of colorectal cancer patients (CR30).

	Global health status/QoL			Physical functioning			Role functioning			Emotional functioning			Cognitive functioning			Social functioning		
	B	95% CI	*p*-value	B	95% CI	*p*-value	B	95% CI	*p*-value	B	95% CI	*p*-value	B	95% CI	*p*-value	B	95% CI	*p*-value
Age Marital status Education level Employment Income Tumor location	−5.332 2.014 2.704 −2.039 2.830 −1.553	−11.22–55 −4.18–8.21 −2.02–7.43 −6.13–2.05 −3.38–9.04 −11.20–8.09	0.075 0.521 0.259 0.325 0.369 0.750	−6.352 0.080 2.926 −2.599 5.428 4.883	−13.33–0.63 −7.27–7.43 −2.68–8.53 −7.45–2.25 −1.94–12.80 −6.56–16.33	0.074 0.983 0.303 0.291 0.147 0.400	−6.800 −0.652 2.569 −4.549 −8.326	−15.35–1.75 −9.66–8.36 −4.29–9.44 −10.43 –1.39 −9.20–8.86 −5.69–22.35	0.118 0.886 0.460 0.132 0.970 0.242	−2.631 −114 −1.802 2.004 6.253 6.280	−9.38 –4.12 −7.22 –6.99 −7.22–3.62 −2.68–6.69 −.87–13.38 −4.78–17.35	0.441 0.975 0.511 0.399 0.085 0.263	−4.678 −3.071 −1.789 −1.299 8.746 5.307	−10.56–1.21 −9.27–3.13 −6.51–2.94 −5.39–2.79 2.53–14.96 −4.34–14.96	0.118 0.328 0.455 0.530 0.006 0.278	−8.141 −1.970 .990 −.176 −.351 .751	−15.88–0.40 −10.12–6.18 −5.22–7.20 −5.55–5.20 −8.52–7.82 −11.94–13.44	0.039 0.633 0.753 0.948 0.932 0.907

	Fatigue			Nausea and vomiting			Pain			Dyspnea			Insomnia			Appetite loss		
	B	95% CI	*p*-value	B	95% CI	*p*-value	B	95% CI	*p*-value	B	95% CI	*p*-value	B	95% CI	*p*-value	B	95% CI	*p*-value

Age Marital status Education level Employment Income Tumor location	4.047 .369 −2.135 2.650 −3.816 1.255	−3.49–11.59 −7.57–8.31 −8.19–3.92 −2.59–7.89 −11.77–4.14 −11.10–13.61	0.290 0.927 0.486 0.318 0.344 0.841	1.853 3.291 5.460 −2.611 −10.672 −5.796	−4.82–8.53 −3.74–10.32 .10–10.82 −7.25–2.03 −17.72–−3.63 −16.74–5.14	0.583 0.355 0.046 0.267 0.003 0.296	5.547 2.168 −1.168 .789 −4.792 −748	−2.04–13.13 −5.82 –10.16 −7.26–4.92 −4.48–6.06 −12.80–3.22 −13.18–11.69	0.150 0.592 0.705 0.767 0.238 0.905	1.982 4.188 2.439 -2.787 -3.145 -3.017	−5.29–9.25 −3.47–11.84 −3.40–8.27 −7.84–2.26 −10.82–4.53 −14.93–8.90	0.590 0.281 0.409 0.276 0.418 .617	−2.879 4.243 1.330 −353 −8.970 −4.390	−11.82–6.06 −5.17–13.66 −5.8–8.51 −6.5–5.86 −18.41–0.47 −19.05–10.27	0.525 0.374 0.714 0.911 0.062 0.554	7.604 −1.202 1.953 −3.261 −6.164 −4.152	−0.79–15.99 −10.04–7.63 −4.78–8.69 −9.09–2.57 −15.02–2.69 −17.91–9.60	0.075 0.788 0.67 0.270 0.171 0.551

	Constipation			Diarrhea			Financial difficulties											
	B	95% CI	*p*-value	B	95% CI	*p*-value	B	95% CI	*p*-value									

Age Marital status Education level Employment Income Tumor location	6.217 −4.394 8.122 −5.593 −12.125 −3.136	−2.15–14.58 −13.20–4.41 1.41–14.84 −11.40–0.22 −20.95–-3.30 −16.85–10.57	0.144 0.325 0.018 0.059 0.008 0.651	−123 9.045 1.574 −1.677 2.363 −2.072	−8.63–8.39 0.08–18.01 −5.26–8.40 −7.59–4.24 −6.62–11.35 −16.02–11.88	0.977 0.048 0.649 0.575 0.603 0.769	1.596 –10.322 –2.017 1.039 –10.444 2.672	–6.65–9.85 –19.01–1.63 –8.64–4.61 –4.69–6.77 –19.15–1.73 –10.85–16.20	0.702 0.020 0.547 0.720 0.019 0.696									

**Table 4:** Predictors of quality of life of colorectal cancer patients (CR29).

	Body image			Anxiety			Weight			Sexual interest			Urinary frequency			Blood and mucus in stool		
	B	95% CI	*p* value	B	95% CI	*p* value	B	95% CI	*p* value	B	95% CI	*p* value	B	95% CI	*p* value	B	95% CI	*p* value
Age Marital status Education level Employment Income Tumor location	5.503 −2.919 .025 1.053 −3.455 −4.064	−2.31–13.32 −11.15–5.31 −6.25–6.30 −4.38–6.48 −11.71–4.80 −16.88–8.75	0.166 0.484 0.994 0.702 0.409 0.531	4.266 −11.682 −0.685 −3.503 −6.026 −3.475	−4.53–13.07 −20.95–-2.41 −7.75–6.38 −9.62–2.61 −15.32–3.26 −17.90–10.95	0.339 0.014 0.848 0.259 0.201 0.634	1.640 −3.035 3.922 −2.692 −7.175 −7.113	−7.00–10.28 −12.13–6.06 −3.01–10.86 −8.70–3.31 −16.30–1.95 −21.28–7.05	0.708 0.510 0.265 0.376 0.122 0.322	−8.051 −12.935 −2.065 3.309 1.322 7.929	−17.70–1.60 −28.21–2.34 −10.39–6.26 −4.67–11.29 −8.92–11.56 −10.78–26.64	0.100 0.095 0.621 0.410 0.797 0.400	1.554 0.771 −6.545 5.248 5.853 −1.109	−6.10–9.21 −7.29–8.83 −12.69–-0.40 −0.07–10.56 −2.22–13.93 −13.65–11.43	0.688 0.850 0.037 0.053 0.154 0.861	1.884 0.417 4.964 −1.621 −8.246 4.896	−3.80–7.56 −5.56–6.40 0.41–9.52 −5.57–2.32 −14.24–-2.25 −4.41–14.21	0.512 0.890 0.033 0.417 0.007 0.300

	Urinary incontinence			Dysuria			Abdominal pain			Buttock pain			Bloating			Dry mouth		
	B	95% CI	*p* value	B	95% CI	*p* value	B	95% CI	*p* value	B	95% CI	*p* value	B	95% CI	*p* value	B	95% CI	*p* value

Age Marital status Education level Employment Income Tumor location	6.161 −5.700 −1.371 1.663 −3.203 7.985	−0.19–12.51 −12.39–0.99 −6.47–3.73 −2.75–6.08 −9.91–3.50 −2.43–18.40	0.057 0.094 0.595 0.457 0.346 0.131	2.522 −3.069 −4.721 1.936 3.084 12.758	−4.10–9.15 −10.05–3.91 −10.04–0.60 −2.67–6.54 −3.91–10.08 1.89–23.62	0.452 0.385 0.081 0.407 0.384 022	7.708 −5.719 0.305 −4.247 −3.463 −1.202	−0.80–16.21 −14.67–3.24 −6.52–7.13 −10.15–1.66 −12.44–5.51 −15.14–12.74	0.075 0.208 0.930 0.157 0.446 0.865	2.993 −3.078 3.381 −1.317 −4.578 3.214	−5.62–11.61 −12.15–6.00 −3.54–10.30 −7.30–4.67 −13.68–4.52 −10.91–17.34	0.493 0.503 0.335 0.664 0.321 0.653	6.974 0.051 0.749 −2.946 −8.269 −2.571	−2.80–16.75 −10.94–11.04 −7.12–8.62 −10.36–4.47 −19.19–2.65 −19.52–14.37	0.159 0.993 0.850 0.431 0.136 0.763	−1.623 4.137 1.555 0.335 −0.969 −5.771	−9.36–6.12 −4.01–12.29 −4.66–7.77 −5.04–5.71 −9.14–7.20 −18.46–6.92	0.679 0.317 0.621 0.902 0.815 0.369

	Hair loss			Taste			Fecal incontinence			Sore skin			Impotence			Dyspareunia		
	B	95% CI	*p* value	B	95% CI	*p* value	B	95% CI	*p* value	B	95% CI	*p* value	B	95% CI	*p* value	B	95% CI	*p* value

Age Marital status Education level Employment Income Tumor location	4.338 5.542 6.003 −4.296 −8.297 −10.827	−4.34–13.02 −3.60–14.68 −0.96–12.97 −10.33–1.73 −17.46–0.86 −25.05–3.40	0.324 0.232 0.090 0.161 0.075 0.134	5.197 2.869 4.558 −2.510 −4.255 −11.884	−2.24–12.64 −4.97–10.71 −1.42–10.53 −7.68–2.66 −12.11–3.60 −24.08–0.32	0.169 0.470 0.133 0.338 0.286 0.056	2.616 0.937 1.578 2.865 -8.715 4.682	−5.75–10.98 −8.46–10.34 −5.15–8.31 −3.47–9.20 −18.05–0.62 −9.81–19.17	0.535 0.843 0.642 0.370 0.067 0.522	7.093 −1.968 7.115 −4.386 −12.137 4.259	−2.58–16.77 −12.76–8.82 −0.65–14.88 −11.66–2.89 −22.86–−1.42 −12.38–20.90	0.148 0.717 0.072 0.233 0.027 0.611	−6.911 −5.245 2.860 1.321 2.137 −.527	−13.47–−0.35 −11.69–1.20 −3.21–8.93 −3.23–5.87 −6.57–10.84 −11.99–10.94	0.039 0.108 0.348 0.562 0.624 0.927	2.666 −17.439 5.530 0.695 −7.530 8.019	−9.74–15.07 −29.68–−5.19 −5.96–17.01 −8.18–9.57 −24.06–9.00 −13.67–29.71	0.667 0.006 0.338 0.876 0.364 0.461

## Data Availability

The pertaining data are available with the corresponding author on demand.
